# Extended clinical phenotypes and long-term outcomes of phosphoglucomutase-3 deficiency

**DOI:** 10.70962/jhi.20250176

**Published:** 2025-12-12

**Authors:** Chen Wang, Meera Patel, Amanda Urban, Celeste G. Nelson, Thomas DiMaggio, Theo Heller, Edward W. Cowen, Heidi H. Kong, Heather A. Kenney, Enrico Santangeli, Yanhan Wang, Crhistian Toribio-Dionicio, Nicole Akar-Ghibril, Mehdi Adeli, Corina E. Gonzalez, Steven M. Holland, Luigi D. Notarangelo, Joshua D. Milner, Jonathan J. Lyons, Alexandra F. Freeman

**Affiliations:** 1 Laboratory of Clinical Immunology and Microbiology, National Institute of Allergy and Infectious Diseases, National Institutes of Health, Bethesda, MD, USA; 2 Clinical Research Directorate, Frederick National Laboratory for Cancer Research, Frederick, MD, USA; 3 Laboratory of Allergic Diseases, National Institute of Allergy and Infectious Diseases, National Institutes of Health, Bethesda, MD, USA; 4 Translational Hepatology Section, Liver Diseases Branch, National Institute of Diabetes and Digestive and Kidney Diseases, Bethesda, MD, USA; 5 Dermatology Consultation Service, Dermatology Branch, National Institute of Arthritis and Musculoskeletal and Skin Diseases, National Institutes of Health, Bethesda, MD, USA; 6 Cutaneous Microbiome and Inflammation Section, Dermatology Branch, National Institute of Arthritis and Musculoskeletal and Skin Diseases, National Institutes of Health, Bethesda, MD, USA; 7 Division of Allergy & Immunology, Department of Medicine, University of California San Diego, Veterans Affairs San Diego Healthcare System, La Jolla, CA, USA; 8 Division of Immunology, Allergy and Rheumatology, Joe DiMaggio Children’s Hospital, Memorial Healthcare System, Hollywood, FL, USA; 9 Division of Pediatric Allergy and Immunology, Sidra Medicine/Hamad Medical Corporation, Doha, Qatar; 10 Immune Deficiency Cellular Therapy Program, National Cancer Institute, National Institutes of Health, Bethesda, MD, USA; 11 Department of Pediatrics, Columbia University Irving Medical Center, New York, NY, USA

## Abstract

Longitudinal analysis of a PGM3-deficient cohort reveals a broad clinical spectrum with immune dysregulation and multi-organ dysfunction, including variable neurodevelopmental delays and malignancies. Hematopoietic stem cell transplantation appears promising for atopy and infection susceptibility and requires further study.

## Teaser

PGM3 deficiency is a multisystem disorder characterized by recurrent infections, chronic severe neutropenia, and virus-associated malignancies, often leading to premature mortality in early adulthood. These features strongly support early consideration of hematopoietic stem cell transplantation in affected individuals.

Phosphoglucomutase-3 (PGM3) is an essential enzyme for the biosynthesis of uridine diphosphate *N*-acetylglucosamine, a key building block for diverse protein glycosylation processes. Biallelic hypomorphic *PGM3* mutations cause a rare autosomal recessive congenital disorder of glycosylation with a spectrum of clinical phenotypes, including T-B-NK + severe combined immunodeficiency with or without syndromic features, combined immunodeficiency, or primary atopic disorder (PAD) (1, 2, 3). As more patients have been recognized, it is clear that clinical findings can vary significantly at presentation and evolve over time, even among individuals within the same kindred. However, due to the rarity of this disorder, most data are from isolated case studies. Reported clinical features and management are often influenced by the evaluating specialty and limited by the short follow-up duration.

In this study, we summarized the clinical phenotypes, immunologic findings, treatment, and outcomes of 10 patients (P) with PGM3 deficiency, all enrolled in prospective natural history protocols (NCT00001355 and NCT00006150). P3 and P4 were also enrolled in a small treatment protocol investigating supplemental monosaccharide and nucleoside derivatives in congenital disorders of glycosylation (NCT02511041). All protocols were approved by the Institutional Review Board of the National Institute of Allergy and Infectious Diseases (NIAID), National Institutes of Health (NIH), and all patient and/or their guardians provided informed consent and/or assent as appropriate. P1–7 were reported before (1, 2); however, this study includes additional phenotypic, therapeutic, and long-term follow-up data.

10 patients (7 males and 3 females), from four unrelated kindreds, are included (Table 1). All underwent whole-exome or -genome sequencing, which identified homozygous or compound heterozygous *PGM3* variants, confirmed by Sanger sequencing. We previously established a flow cytometric assay using fluorescein-conjugated plant lectin (i.e., L-phytohemagglutinin [L-PHA]) to assess the surface expression of complex branched *N*-glycans, which showed reduced surface staining on naïve CD4^+^ T cells (CD4^+^CD45RO^−^) from all patients in kindred I and II (i.e., P1–7) (4). This assay revealed reduced L-PHA staining on naïve CD4^+^ T cells in P8–10 compared to healthy controls. The median age at first evaluation was 8 years (range 9 mo–22 years) with the initial phenotype suggestive of PAD in all patients, as evidenced by atopy, elevated serum total immunoglobulin (Ig)E levels, and recurrent sinopulmonary infections.

**Table 1.** Clinical features of patients with PGM3 deficiency

P#	Kindred code	Age at last follow-up, sex	*PGM3* variants	Atopy	Oto-sino-pulmonary infections	Mucocutaneous viral infections	Neurocognitive impairments	Skeletal abnormality	Chronic neutropenia	Clinical autoimmunity	Malignancy	Others	NIH HIES score	HSCT	Outcomes
1	I.1^a^	47, M	Compound heterozygous, c.1585G>C, p.Glu529Gln; c.1438_1442del, p.Leu480SerfsTer10	Atopic dermatitis	Recurrent, c/b bronchiectasis	No	Yes	Scoliosis, degenerative disc disease	No	MPGN (failed kidney transplantation, on dialysis), cutaneous leukocytoclastic vasculitis	No	Nonischemic cardiomyopathy	36	No	Alive
2	I.2	41, F		Atopic dermatitis, food allergy	Recurrent, c/b bronchiectasis	No	Yes	Scoliosis, degenerative disc disease	Yes	Cutaneous leukocytoclastic vasculitis	EBV-positive DLBCL	Chronic norovirus infection	39	No	Died
3	I.3	37, M		Atopic dermatitis, food allergy, eosinophilic esophagitis	Recurrent, c/b bronchiectasis	Molluscum contagiosum, HSV-1 infection (skin, mouth, and eye)	Yes	Scoliosis	Yes	Cutaneous leukocytoclastic vasculitis	EBV-positive Hodgkin lymphoma, DLBCL	​	43	No	Died
4	I.4	42, M		Atopic dermatitis, food allergy, eosinophilic esophagitis	Recurrent, c/b bronchiectasis	Molluscum contagiosum, HSV-1 infection (skin)	Yes	Scoliosis	No	MPGN, cutaneous leukocytoclastic vasculitis	EBV-positive Hodgkin lymphoma; right eye SCC	​	40	No	Died
5	II.1	24, M	Homozygous, c.975T>G, p.Asp325Glu	Atopic dermatitis, food allergy, allergic bronchopulmonary aspergillosis	Recurrent, c/b bronchiectasis	No	Yes	Scoliosis	Yes (with positive anti-neutrophil antibody)	Psoriasis	No	​	42	No	Alive
6	II.2	18, M		Atopic dermatitis, food allergy	Recurrent	Verruca plana	Yes	Scoliosis	Yes	No	No	Coombs-negative hemolytic anemia	38	No	Died
7	II.3	13, M		Atopic dermatitis, food allergy, allergic fungal otomastoiditis	Recurrent	HSV-1 (mouth)	Yes	No	Yes	No	No	​	33	Yes	Alive
8	III.1	11, F	Homozygous, c.982G>A, p.Ala328Thr	Atopic dermatitis, food allergy	Recurrent	No	No	No	Yes	No	No	​	8	No	Alive
9	IV.1	6, M	Compound heterozygous, c.1592C>T, p.Ala531Val; c.620C>G, p.Ser207Ter	Atopic dermatitis, food allergy	No	Molluscum contagiosum	No	End-plate irregularity	No	No	No	​	12	No	Alive
10	IV.2	8, F		Atopic dermatitis, food allergy	Recurrent	Molluscum contagiosum	No	End-plate irregularity	Yes	No	No	​	16	Yes	Alive

M, male; F, female; c/b, complicated by; MPGN, membranoproliferative glomerulonephritis; DLBCL, diffuse large B cell lymphoma; SCC, squamous cell carcinoma; HIES, hyper-IgE syndrome.

a Kindred I includes five clinically affected individuals, with I.3 and I.4 being identical twins. One of them (i.e., the eldest daughter), who was labeled as I.5 in our prior publication (2), had a compatible clinical phenotype based on outside records. She deceased at age 27 before our evaluation of the family. As no genetic material was available to confirm the *PGM3* variants, she is not included in the current table.

All patients had clinical features distinct from *STAT3* dominant-negative hyper-IgE syndrome, DOCK8 deficiency, or other PADs. Retained primary teeth and characteristic facial features were absent from all patients. Although five patients showed varying degrees of bronchiectasis, none developed pneumatocele or bronchopleural fistula. Only one patient had a history of mild mucocutaneous candidiasis, but recurrent viral mucocutaneous infections were observed in six patients (three of four kindreds), including molluscum contagiosum in four. Clinical autoimmunity was observed in five patients (two kindreds), including biopsy-proven cutaneous leukocytoclastic vasculitis in four, membranoproliferative glomerulonephritis in two, and psoriasis in one. Four patients, including two kindreds, had type 2 immunity-related eosinophilic disorders, including eosinophilic esophagitis (P3 and P4), allergic bronchopulmonary aspergillosis (P5), and allergic fungal otomastoiditis (P7). Other findings included skeletal abnormalities, end-stage renal disease with failed kidney transplantation requiring hemodialysis, and heart failure. Malignancy affected three patients in one kindred, including two with Epstein-Barr virus (EBV)–positive nodular sclerosing Hodgkin lymphoma in young adulthood (P3 and P4), two with EBV-positive diffuse large B cell lymphoma during follow-up (P2 and P3), and one with right eye squamous cell carcinoma (P4, unknown human papillomavirus status of the tumor). Neurocognitive impairment, manifested by developmental delay with psychomotor retardation and/or hypomyelination, was present in all seven patients from the first two kindreds (P1–7). In contrast, the remaining three patients from two kindreds met neurodevelopmental milestones and had normal neurological examinations as well as brain magnetic resonance imaging.

Laboratory evaluation showed cytopenias, most notably chronic neutropenia, which was severe in six patients (absolute neutrophil count [ANC] <500 cells/μl) and moderate in one (ANC 500–1,000 cell/μl). Additionally, P6 experienced recurrent episodes of Coombs-negative hemolytic anemia requiring transfusion support. Increased osmotic fragility of red blood cells was demonstrated in P6; however, an extensive evaluation was unremarkable. No culprit medications were identified, and results were normal for red blood cell enzyme panel, hemoglobin electrophoresis, and red blood cell surface band 3. Screening for paroxysmal nocturnal hemoglobinuria by flow cytometry was also negative. Bone marrow biopsies were performed in eight patients with variable findings. Of the five patients with peripheral neutropenia at the time of biopsy, two (P9 and P10) had a markedly hypocellular marrow with myeloid hypoplasia but no dysplastic features or excess blasts, while the others showed normocellular marrow with normal myeloid maturation and without a reactive increase in neutrophil production. P6 showed hypercellular marrow with erythroid hyperplasia, attributed to active peripheral hemolysis. Others had normal bone marrow findings. There were normal levels of serum IgG and IgM in most instances, but IgA levels were elevated in nine patients. In addition, nine had lymphopenia, affecting both T (especially naive subsets) and B cells (especially memory subsets). We assessed serial measurements of peripheral lymphocyte subsets before hematopoietic stem cell transplantation (HSCT) and without repeated systemic corticosteroid use. Patients from kindred 1 showed stable findings over time, whereas P5 showed progressive declines of CD4^+^ T cells. EBV viremia was detected in seven patients (intermittent, *n* = 3; persistent, *n* = 4). Given the high burden of various viral diseases in our cohort, we screened all patients for plasma neutralizing anti-type I interferon (-α, -β, and -ω) autoantibodies using a multiplex particle-based assay; all were negative. Lastly, although previous *N*-linked glycan analysis revealed under-galactosylation in several patients, serum transferrin isoform-based analysis showed normal glycosylation patterns in P8 and P10, consistent with our prior findings in kindred I and II (2).

Assessing management, we noted that, in addition to conventional antimicrobial therapies, five patients received Ig replacement therapy with variable impact on infections. Three were treated with subcutaneous granulocyte colony-stimulating factor for chronic severe neutropenia, resulting in modest-moderate improvement. Four patients received type 2 immunity-directed biologics: dupilumab in three for atopic dermatitis, and omalizumab in one for allergic fungal otomastoiditis. Strikingly, three experienced acute hypersensitivity reactions to the first dose, two to dupilumab and one to omalizumab, the latter requiring intensive care unit admission for anaphylaxis.

One patient underwent bilateral lung transplantation for chronic respiratory failure due to severe bronchiectasis (P2, age 28). Two patients (P7 and P10, both at age 8) underwent allogeneic HSCT with improvement of most immune-related manifestations, including correction of their chronic severe neutropenia. Moreover, both showed normal L-PHA staining on their CD4^+^ T cells after HSCT.

Based on some successful experiences of monosaccharide supplementation in other congenital disorders of glycosylation (5) and our observation that *N*-acetylglucosamine supplementation restored intracellular levels of sugar nucleotides in PGM3-deficient cells in vitro (2), we initiated a trial of sequential oral *N*-acetylglucosamine (maximal dose 100 mg/kg/day) and uridine 5-monophosphate (maximal dose 50 mg/kg/day). P3 received the treatment for ∼5 mo but then developed recurrent abdominal pain and a flare of his cutaneous vasculitis. P4 was on therapy for about 6 mo but showed worsening proteinuria in the context of preexisting membranoproliferative glomerulonephritis (24-h urine protein quantification increased from 1.66 to 3.00 g). As a result, both patients eventually discontinued the treatment, and the trial was halted.

Over a median follow-up of 16 years (range 1–25 years), four patients died. Causes of death included EBV-positive diffuse large B cell lymphoma (P2, age 41; P3, age 37), metastatic squamous cell carcinoma (P4, age 42), and respiratory failure due to acute coronavirus disease 2019 (COVID-19) infection (P6, age 18). P6, who died from COVID-19, was wheelchair bound due to the severe progressive neurologic decline and chronic multifactorial respiratory failure. The overall survival at ages 20 and 40 years was 83% and 63%, respectively.

PGM3 deficiency presents with a broad spectrum of clinical manifestations involving both immune and nonimmune systems. By leveraging a standardized clinical protocol that enabled comprehensive evaluation and longitudinal follow-up, we described phenotypic variability in 10 patients, the largest single-center cohort reported to date. Despite best supportive care, disease burden accumulated over time, particularly with the emergence of malignancies, including EBV-positive lymphoma, leading to premature adult mortality. The heterogeneity in clinical phenotypes and disease progression is likely influenced by residual PGM3 enzyme activity, genetic background, and environmental exposures. While HSCT offers a potential cure for immune defects, its impact on nonimmune manifestations remains less studied. We also attempted a sugar-amine supplementation protocol predicted to improve the defective glycosylation pathway. The enhanced inflammation observed, potentially reflecting improved immune cell effector function, precluded further study and necessitates additional preclinical work to optimize this approach for PGM3 deficiency. Our study is limited by the absence of patients with either severe combined immunodeficiency or combined immunodeficiency without PAD features, likely due to referral bias for phenotypes associated with atopy.

In summary, although PGM3 deficiency is a multisystemic disorder, hallmark features of recurrent infections, chronic severe neutropenia, and virus-driven malignancies with early adult mortality strongly support early HSCT consideration in these patients.

## Data Availability

All data are either included in the manuscript or available upon reasonable request.
